# Native bacteria isolated from roots and rhizosphere of *Solanum lycopersicum L*. increase tomato seedling growth under a reduced fertilization regime

**DOI:** 10.1038/s41598-020-72507-4

**Published:** 2020-09-24

**Authors:** María Micaela Pérez-Rodriguez, Patricia Piccoli, María Soledad Anzuay, Rita Baraldi, Luisa Neri, Tania Taurian, Miguel Andrés Lobato Ureche, Diana María Segura, Ana Carmen Cohen

**Affiliations:** 1 Instituto de Biología Agrícola de Mendoza (IBAM-CONICET), Facultad de Ciencias Agrarias, Universidad Nacional de Cuyo, 5507 Chacras de Coria, Mendoza Argentina; 2 Departamento de Ciencias Naturales, Facultad de Ciencias Exactas, Físico-Químicas y Naturales, Universidad Nacional de Río Cuarto, Agencia Postal 3, 5800 Río Cuarto, Córdoba Argentina; 3 National Research Council of Italy, Institute of BioEconomy (CNR-IBE), via P. Gobetti 101, 40129 Bologna, Italy

**Keywords:** Soil microbiology, Applied microbiology

## Abstract

In semiarid regions is important to use native strains best adapted to these environments to optimize plant-PGPR interaction. We aimed to isolate and characterize PGPR from roots and rhizosphere of a tomato crop, as well as studying the effect of its inoculation on tomato seedlings growth. We selected four strains considering their effectiveness of fixing nitrogen, solubilizing phosphate, producing siderophores and indole acetic acid. They belong to the genera *Enterobacter*, *Pseudomonas*, *Cellulosimicrobium,* and *Ochrobactrum*. In addition, we also analyzed the ability to solubilize Ca_3_(PO_4_)_2_, FePO_4_ and AlPO_4_ and the presence of one of the genes encoding the cofactor PQQ in their genome. *Enterobacter* 64S1 and *Pseudomonas* 42P4 showed the highest phosphorus solubilizing activity and presence of *pqq*E gene. Furthermore, in a tomato-based bioassay in speed-bed demonstrated that a sole inoculation at seedling stage with the strains increased dry weight of roots (49–88%) and shoots (39–55%), stem height (8–13%) and diameter (5–8%) and leaf area (22–31%) and were equal or even higher than fertilization treatment. Leaf nitrogen and chlorophyll levels were also increased (50–80% and 26–33%) compared to control. These results suggest that *Enterobacter* 64S1 and *Pseudomonas* 42P4 can be used as bio-inoculant in order to realize a nutrient integrated management.

## Introduction

Conventional agriculture is mostly dependent on chemical fertilizers and pesticides^1^. Agrochemicals are used to increase agricultural productivity in order to feed the increasing world population. These agronomic practices are expensive and they led to a degradation of agricultural lands and consequently produce negative impacts in the environment, especially if overused^2, 3^. The major inconveniences are groundwater contamination and eutrophication of surface waters^4, 5^. The plants can assimilate only a 30–40% of the nitrogen (N) applied despite the overuse of N fertilizers^5, 6^. After N, phosphorus (P) is the second plant growth-limiting macronutrient. A significant percentage of this element is in insoluble form and only a small proportion is available for plants^7^. Furthermore, soluble P is highly reactive with soil Ca^2+^, Fe^2+^ or Al^3+^, leading to its precipitation, so fertilization efficiency is low^7, 8^. Therefore, the use of plant growth-promoting rhizobacteria (PGPR) in agriculture could be a sustainable and environmentally friendly solution reducing problems associated with the overuse of chemicals fertilizers^9^. PGPR stimulate plant growth through several mechanisms. Direct promotion includes the enhanced nutrient availability and nutrient use efficiency, as well as the ability to solubilize P, to fix N_2_, to produce siderophores, deaminase activity (1-aminocyclopropane-1-carboxylate) and of producing plant hormones such as, abscisic acid, gibberellins, indole-3-acetic acid (IAA)^10–12^, among others. The solubilization of mineral P is related to bacterial secretion of low molecular weight organic acids, mainly gluconic and 2-cetogluconic acids^13^. The pyrroloquinoline quinone (PQQ) cofactor is necessary for the activity of glucose dehydrogenase (GDH) that catalyses the oxidation of glucose to gluconic acid. The biosynthesis of PQQ cofactor involves a PQQ operon consisting of at least 5–7 genes^14, 15^. Thus, a *pqqE* gene encoding PQQ is involved in phosphorus solubilization^14^. Moreover, indirect promotion includes protection of the plant against pathogenic agents, acting as biocontrol bacteria or inducing tolerance to stress^9, 16, 17^. PGPR are common inhabitants of the soil, but their number is not enough to compete with the other bacteria established in the rhizosphere. Therefore the inoculation of PGPR is necessary to increase the soil number of target microorganism and maximize their beneficial properties for plant yield^13^. The use of native soil bacterial isolates has the advantage of easier adaptation and success when inoculated into the plant rhizosphere^18^. In addition, they are more resistant to local environmental stresses especially under the predicted climatic changes scenarios^19–21^.

Tomato is the second most produced vegetable crop in the world (The Food and Agriculture Organization of the United Nations, FAO). Argentina occupies the 12^th^ position in the world production. In the 2018–2019 seasons this crop reached 395,000 tons in 5,514 ha. Cuyo region (central-west Argentina) has a high impact on the industrial tomato, contributing with 78.7% of the total national production. Mendoza, located in a semi-arid region, is one of the main industrial tomato producers in the Cuyo region, but the national demand is not satisfied by the local production^22^. To increase the production and quality in the tomato industry large amount of chemical fertilizers are required, with all of negative consequences mentioned above. However, it is necessary to increase the production in a sustainable way. Although there are several studies regarding PGPR isolated from other environments and their effect on tomato^23–25^, information regarding native PGPR isolates from tomato crop in Mendoza is scarce.

The aim of this work was to isolate and characterize PGPR from roots and rhizosphere of tomato crops and to study the effect of their inoculation on the growth of tomato seedlings in order to reduce the fertilizer’s rates and produce high quality plantlets (with an increased root system) that are suitable for transplanting into the farm.

## Results

### Bacteria isolation and screening for the nitrogen fixation ability, phosphate solubilization and siderophores production

A total of 90 bacteria were isolated from tomato roots and rhizosphere (40 and 50, respectively). Then, they were screened for N_2_ fixation ability and from them, 36 isolated were able to grow in N-free media (50% from rhizosphere and 50% from roots), (Table 1). The majority of the bacterial isolates showed bacillus shape (75%) and Gram negative (72%). Then, phosphate solubilization capacity and production of siderophores of the 36 isolates with N_2_ fixation ability were analyzed. Out of the 36 isolates, 33 showed a clear phosphate solubilization zone around the colony. The isolates 64S1 and 42P4 exhibited the highest phosphate solubilization efficiency (360 and 283%, respectively). Most strains produced siderophores (29 isolates) showing the appearance of orange halo on CAS medium. The higher halo percentage was detected in 42P4 and 25X1 strains (1.33 and 1.30%, respectively). In addition to the three bacterial isolates that exhibited the highest phosphate solubilization efficiency and siderophore production, strains 60I1 and 53F were also selected due to their phosphate solubilization efficiency (207.5 and 217%) and siderophores production (0.8 and 0.75% respectively) (Supplementary Fig. S1 and S2).

**Table 1:** Summary of plant growth promoting (PGP) traits showed by native nitrogen fixing bacterial strains isolated from rhizosphere and roots of tomato crop.

Strain	Source	Shape	Gram reaction^†^	P solubilization efficiency (%)	Siderophores production (% halo)
6R1	Rhiz	Cocci	−	120	nd
6L	Rhiz	Cocci	+	120	nd
22D4	Rhiz	Bacilli	−	200	0.80
25L4	Rhiz	Bacilli	−	140	0.67
27T4	Rhiz	Bacilli	−	260	0.78
28H	Rhiz	Bacilli	+	160	0.20
35I	Rhiz	Bacilli	+	120	nd
40B4	Rhiz	Bacilli	−	120	0.82
45R4	Rhiz	Bacilli	−	140	0.33
42P4	Rhiz	Bacilli	−	283	1.33
42Q4	Rhiz	Bacilli	−	250	0.67
46A	Rhiz	Bacilli	−	nd	nd
46B	Rhiz	Bacilli	−	nd	nd
89B	Rhiz	Cocci	−	120	nd
50G	Rhiz	Cocci	+	180	0.4
60I1	Rhiz	Bacilli	+	207.5	0.8
65I4	Rhiz	Bacilli	−	250	0.5
74M4	Rhiz	Bacilli	−	267	0.67
2O	Root	Cocci	+	120	0.33
8Q1	Root	Cocci	−	156	nd
20L1	Root	Bacilli	−	140	nd
24 K	Root	Bacilli	+	120	0.2
24K1	Root	Bacilli	−	120	0.14
25X1	Root	Bacilli	−	200	1.30
25X2	Root	Bacilli	−	140	0.4
43Y	Root	Bacilli	−	240	0.60
53F	Root	Bacilli	−	217	0.75
53F1	Root	Bacilli	−	167	0.4
54E	Root	Bacilli	−	nd	nd
57B1	Root	Cocci	+	120	nd
59U4	Root	Bacilli	−	167	0.89
59U5	Root	Bacilli	−	200	0.50
62F2	Root	Cocci	+	150	0.8
64H1	Root	Cocci	+	120	nd
64S1	Root	Bacilli	−	360	0.88
67Q	Root	Bacilli	−	120	0.73

^†^( +) positive; (−) negative; *nd* not detected.

### Biochemical characterization of selected bacterial isolates

A selection of five strains was realized according to the best exhibition of PGP traits (Table 2). All isolates were catalase positive and showed capacity to produce ammonium. Wherever, among then, only 42P4 and 53F strains presented cytochrome oxidase activity. Also, protease activity was detected in the strains 42P4, 53F and 64S1. The fastest growth was observed in 64S1 strain, reaching the stationary phase at 7 h (Supplementary Fig. S3), while for 25X1, 42P4 and 53F strains, at 12–14 h. In addition, 60I1 was the slowest strain to get to this phase at 24 h. At different temperatures, the highest concentration of CFUs mL^−1^ was determined at 28 °C in all the strains while the lowest concentration was at 4 °C except for 25X1 that grew less at 40 °C. All the strains produced IAA, the highest concentration of IAA were detected in bacterial culture of the strain 64S1 and the lowest on 25X1 (Table 2).

**Table 2:** Biochemical characteristics of native strains isolated from rhizosphere and roots of tomato crop.

Strain	Catalase^†^	Ammonia production^†^	Cytochrome oxidase^†^	Protease activity^†^	CFU mL^−1^ at different temperature of growing^‡^			Plant hormone IAA (ng mL^–1^)^‡^
					4 °C	28 °C	40 °C	
25X1	+	+	−	−	103 ± 4^b^	159 ± 6^a^	61 ± 6^c^	100.6 ± 9.40^e^
42P4	+	+	+	+	40 ± 8^c^	97 ± 9^a^	68 ± 7^b^	482.4 ± 1.10^b^
53F	+	+	+	+	246 ± 11^c^	463 ± 13^a^	275 ± 8^b^	260.8 ± 25.10^d^
60I1	+	+	−	−	36 ± 6^c^	73 ± 9^a^	54 ± 5^b^	368.8 ± 74.90^c^
64S1	+	+	−	+	63 ± 6^c^	114 ± 12^a^	83 ± 8^b^	2,387.8 ± 28.10^a^

^†^(−) indicates absence of PGP trait; (+) indicates presence of PGP trait.

^‡^Each value is a mean ± S.E. of three independent replicates (n = 3). Different letters indicate differences among isolates according to LSD Fisher test (*P* < 0.05).

### Identification of bacterial isolates

BLAST result indicated that 25X1 strain was 100% similar to *Stenotrophomonas maltophilia* (CP040433.1L); 42P4 strain was 100% similar to *Pseudomonas corrugata* (MK.774793.1), *P. fluorescens* (MF.000304.1), *P. thivervalensis* (KU.500610.1) and *P. brassicacearum* (KT.215482.1); 53F strain was 100% similar to *Ochrobactrum anthropi* (AF.526523.2); 60I1 strain was 99.7% similar to *Cellulosimicrobium cellulans* (NR_115251.1) and *C. funkei* (JQ.659856.1); 64S1 was 99.8% similar to *Enterobacter cloacae* (MG.557804.1) and *E. hormaechei* (KF.254587.1). As *Stenotrophomonas maltophilia* (25X1) may be a pathogen in immunosuppressed patients, we decided not to use the strain 25X1 although its PGPR effect has been reported in *Arachis hypogea*^26^.

To confirm the identity of the isolated strains, phylogenetic analyses were carried out. Seven sequences of each species obtained by BLAST analysis were aligned with the strains of this work, searching if any of them form monophyletic groups. The trees obtained show that only 42P4 formed a monophyletic group with *Pseudomonas brassicacearum* (Supplementary Fig. S4).

### Quantification of soluble phosphate released by selected bacteria

**Figure 1:**
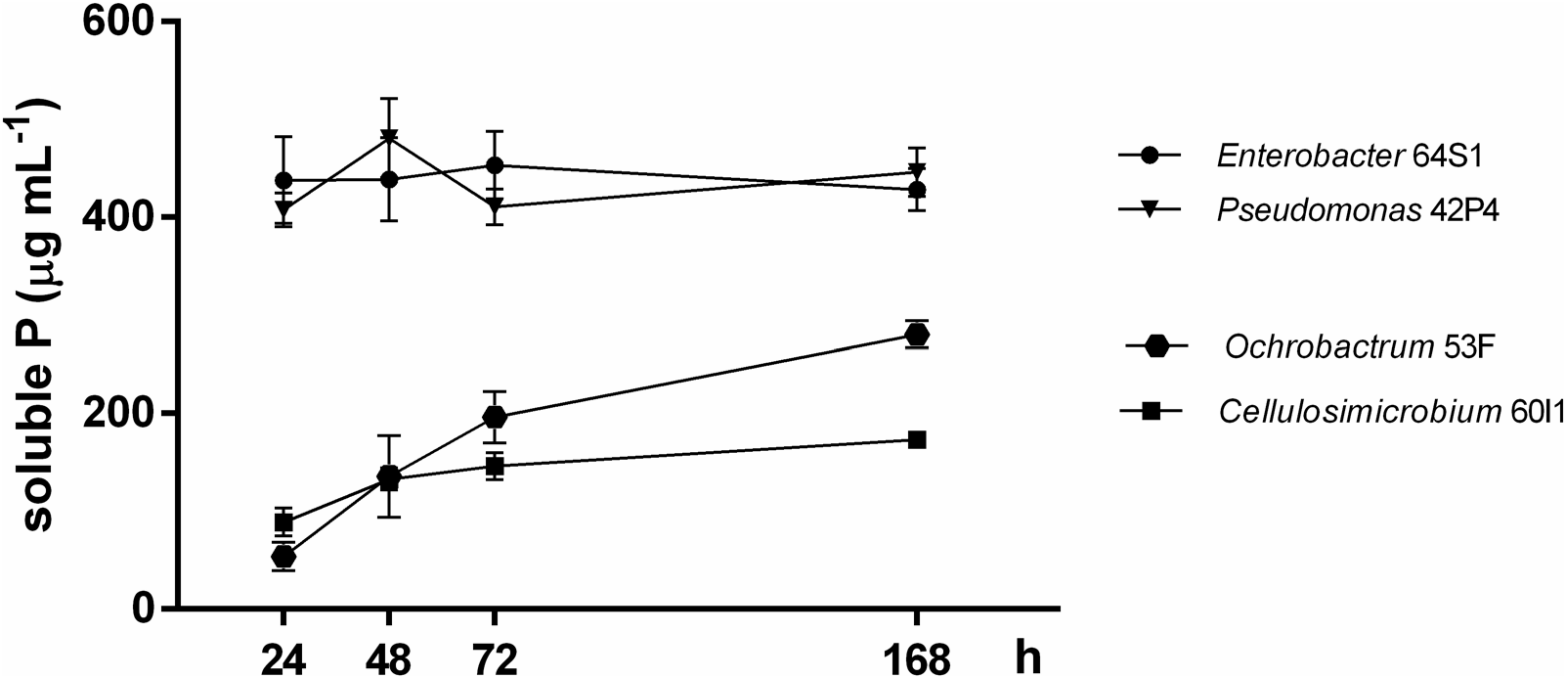
Levels of soluble phosphorus released by *Enterobacter* 64S1, *Pseudomonas* 42P4, *Cellulosimicrobium* 60I1 and *Ochrobactrum* 53F. Data are means ± S.E. of six replicates according to LSD Fisher test (*P* < *0.05*).

The concentration of soluble P released (Pr) by the strains in liquid medium NBRIP-BPB ranged from 173.08 to 437.98 μg mL^−1^ (Table 3). The highest amounts of Pr were detected in *Enterobacter* 64S1 and *Pseudomonas* 42P4 medium (437.98 μg mL^−1^ and 407.54 μg mL^−1^ respectively). By the contrary, the lowest amounts of P were liberated by isolates of *Ochrobactrum* 53F and *Cellulosimicrobium* 60I1 (280.40 μg mL^−1^ and 173.08 μg mL^−1^, respectively). Both, 64S1 and 42P4 cultures showed the highest concentration of soluble Pr at 24 h and remained constant until 168 h (Table 3 and Fig. 1). The pH of all isolates cultures dropped from 7 (initial value) to 3.89–4.52. On the other hand, the numbers of CFU mL^−1^ observed in the isolates indicated that the viability of isolates was not affected along the assay until the end of the experiment (Table 3).

**Table 3:** Maximum amounts of P-released, time of growth, pH and colony forming units (CFU mL^−1^) in NBRIP-BPB media with Ca_3_(PO_4_)_2_ by native bacteria (64S1, 42P4, 60I1 and 53F).

Strain	P released (μg mL^−1^)^†,#^	Time of growth (h)^‡^	pH^§,#^	CFU mL^−1††^
*Enterobacter* 64S1	437.98 ± 44.07^a^	24	3.93 ± 0.03^c^	3 × 10^8^
*Pseudomonas* 42P4	407.54 ± 17.34^a^	24	3.89 ± 0.03^c^	8 × 10^8^
*Cellulosimicrobium* 60I1	173.08 ± 7.72^c^	168	4.52 ± 0.04^a^	4 × 10^7^
*Ochrobactrum* 53F	280.40 ± 13.81^b^	168	4.14 ± 0.05^b^	4 × 10^8^

^†^Maximum levels of soluble phosphorus released.

^‡^Time of growth (h) in which maximum levels of soluble P were released.

^§^Lowest culture pH reached during incubation time.

^††^Colony-forming units (CFU mL^−1^) at time of maximum levels of soluble P released by each bacterium.

^#^Values are mean ± SE of six independent replicates (n = 6). Different letters indicate differences among isolates according to LSD Fisher test (*P* < 0.05).

In NBRIP-BPB solid medium either with AlPO_4_ and FePO_4,_ only *Enterobacter* 64S1 and *Pseudomonas* 42P4 showed high activity of P solubilization. Therefore, the capacity of these strains to be considered as phosphate solubilizing bacteria (PSB) was evaluated. Considering these results, quantitative analysis of phosphate solubilizing capacity of 64S1 and 42P4 in NBRIP-BPB liquid medium with AlPO_4_ and FePO_4_ was developed. For 64S1 with AlPO_4_ and FePO_4_ the Pr concentration reached out the 88.39 μg mL^−1^ and 11.25 μg mL^−1^_,_ respectively. On the other hand, for 42P4, the Pr activity was lesser with AlPO_4_ and major with FePO_4_ (28.16 and 15.10 μg mL^−1^, respectively) compared to 64S1. In presence of the both sources of insoluble phosphorus analyzed the maximum amount of Pr was at 7 days and the viability of isolates was not affected (Table 4).

**Table 4:** Maximum amounts of P-released, pH, colony forming units and time of growth in NBRIP-BPB medium with FePO_4_ and AlPO_4_ by the 64SI and 42P4 strain native isolated from tomato root and rhizosphere.

Strain	Time of growth (h)^†^	NBRIP-BPB medium with AlPO_4_			NBRIP-BPB medium with FePO_4_		
		P released (μg mL^−1^)^‡,#^	pH^§,#^	CFU mL^−1††^	P released (μg mL^−1^)^‡,#^	pH^§,#^	CFU mL^−1††^
*Enterobacter* 64S1	168	88.39 ± 10.19^a^	3.54 ± 0.01^a^	6 × 10^5^	11.25 ± 2.98^a^	3.54 ± 0.19^a^	3 × 10^5^
*Pseudomonas* 42P4	168	28.16 ± 4.37 ^b^	3.68 ± 0.25^a^	5 × 10^6^	15.10 ± 2.56^a^	3.64 ± 0.03^a^	6 × 10^5^

^†^Time of growth (h) in which maximum levels of soluble P were released.

^‡^Maximum levels of soluble phosphorus released.

^§^Supernatants pH at time of maximum levels of soluble P released by each bacterium.

^††^Colony-forming units at time of maximum levels of soluble P released by each bacterium.

^#^Values are means ± S.E., of six independent replicates (n = 6). Different letters indicate differences among isolates according to LSD Fisher test (*P* < 0.05).

### Detection of *pqq*E gene in bacterial genomes

**Figure 2:**
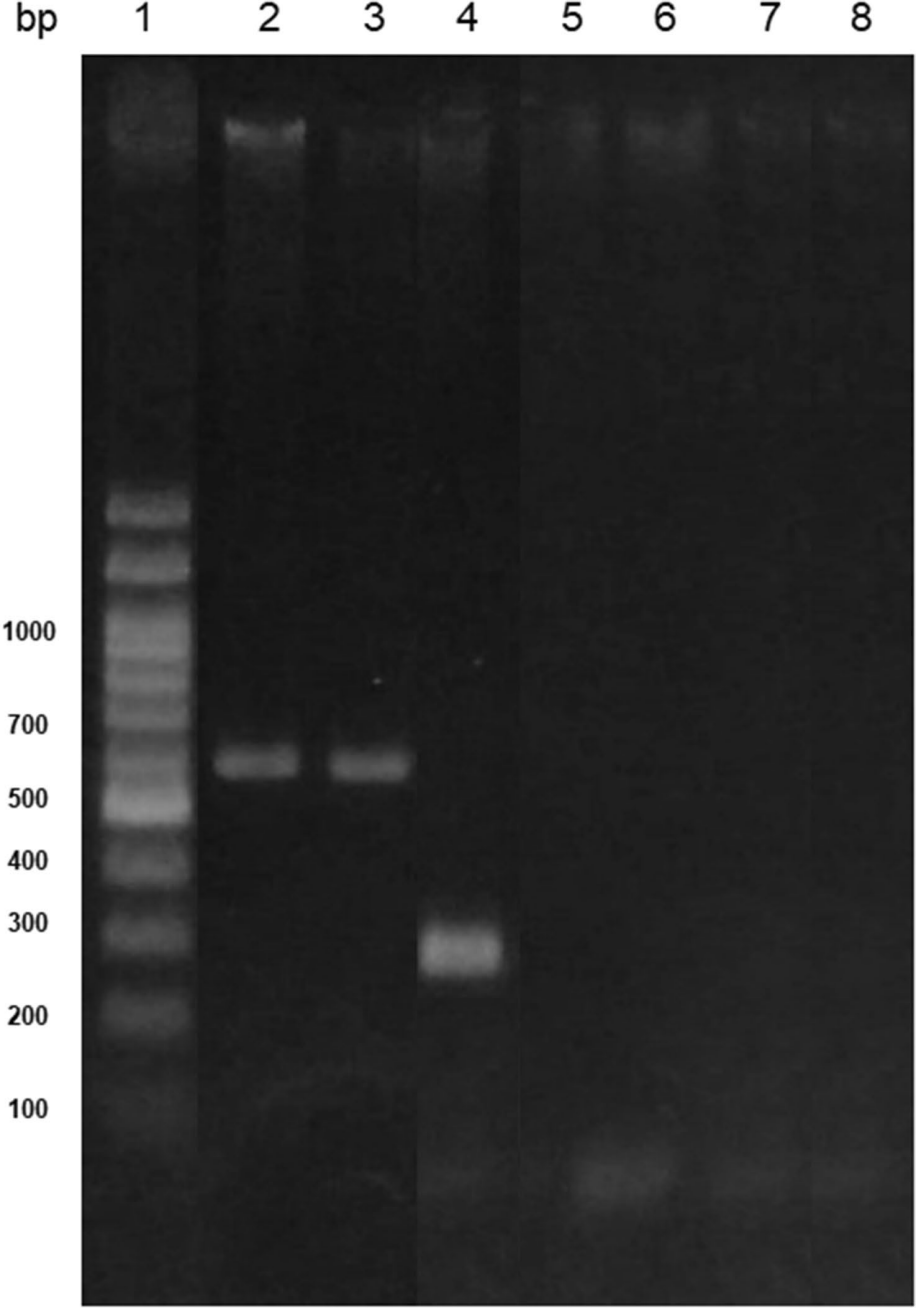
*pqqE*-PCR products of DNA obtained from the strains. Line 1: DNA ladder 100 bp (PBL product); Line 2: *Pseudomonas* 42P4; Line 3: *Enterobacter* 64S1; Line 4: *Enterobacter* 64S1; Line 5: *Cellulosimicrobium* 60I1; Line 6: *Ochrobactrum* 53F; Lines 7 and 8: negative control. Lines 2, 3, 5 and 6 correspond to the amplification products (~ 700 bp) with primers F317 and R1019; Line 4 corresponds to the amplification products (~ 350 bp) with primers *pqq*EENT1 and *pqq*EENT2.

The presence of one of the gene encoding for the cofactor PQQ of GDH enzyme, responsible for gluconic acid production, was evaluated in the genome of bacterial isolates. The amplification of *pqq*E gene fragment was assayed using two sets of primers (F317 and R1019 and *pqq*EENT1 and *pqq*EENT2), that amplify fragments of ~ 700 and ~ 350 bp, respectively. *Enterobacter* 64S1 and *Pseudomonas* 42P4 exhibited a band of the expected size corresponding to the fragment *pqq*E (700 bp) (Fig. 2). PCR amplification product was not observed for *Ochrobactrum* 53F and *Cellulosimicrobium* 60I1. On the other hand, *Enterobacter* 64S1 strain presented a band of ~ 350 bp corresponding to a fragment of *pqq*E gene by using specific primers designed in this work (Fig. 2).

### Effects of inoculation with selected strains on the growth of tomato plantlets with strain selected

**Figure 3:**
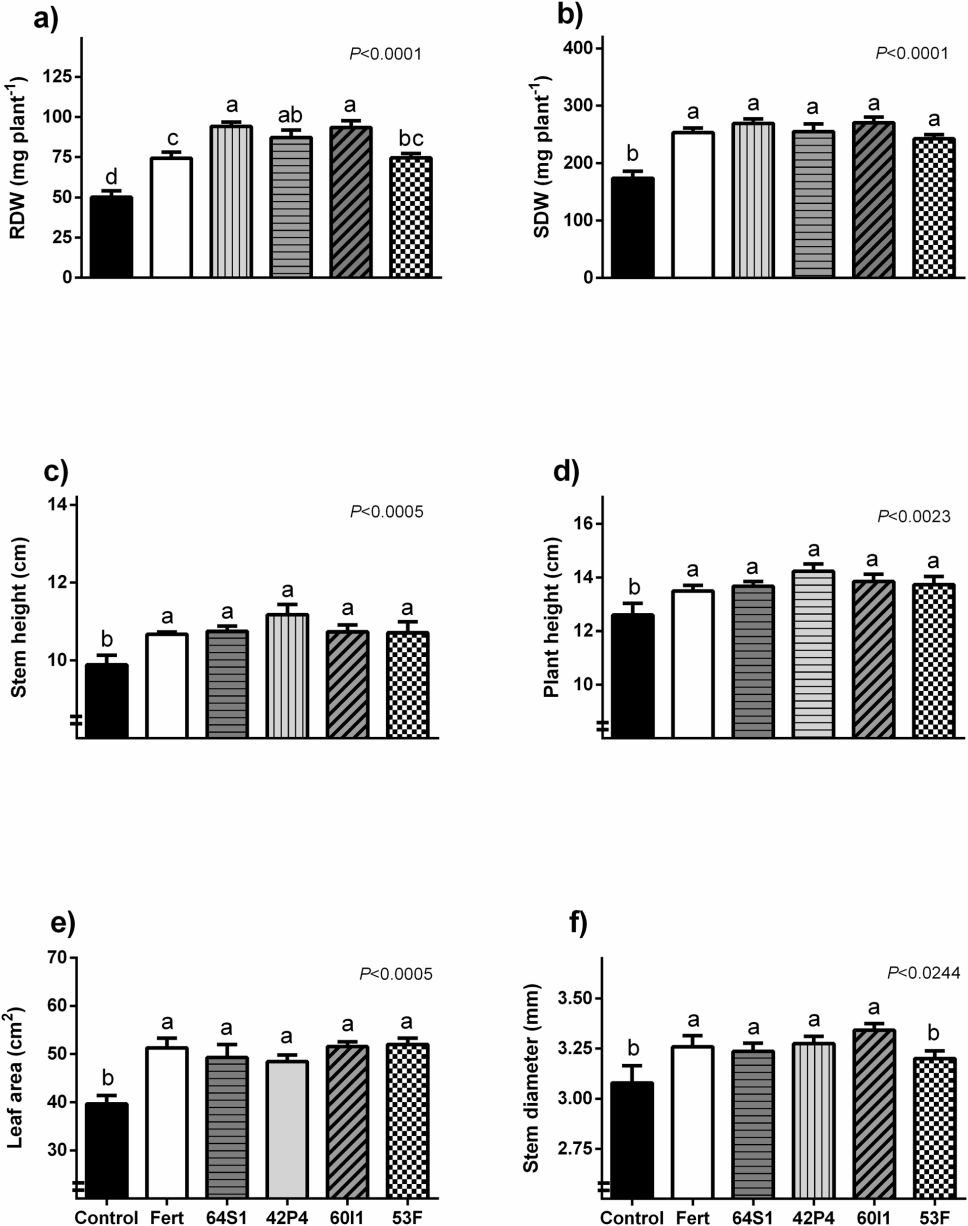
Root and shoot dry weight (mg plant^−1^) (**a**,**b**), stem height (cm) (**c**), plant height (cm) (**d**), foliar area (cm^2^) (**e**) and stem diameter (mm) (**f**) of tomato plantlets inoculated with PSB (Control), Fert: Fertilized treatment, 64S1: *Enterobacter* 64S1, 42P4: *Pseudomonas* 42P4, 60 I1: *Cellulosimicrobium* 60I1 and 53F: *Ochrobactrum* 53F. Data are means ± S.E., of 11 replicates. Different letters indicate differences among isolates according to LSD Fisher test (*P* < 0.05).

**Figure 4:**
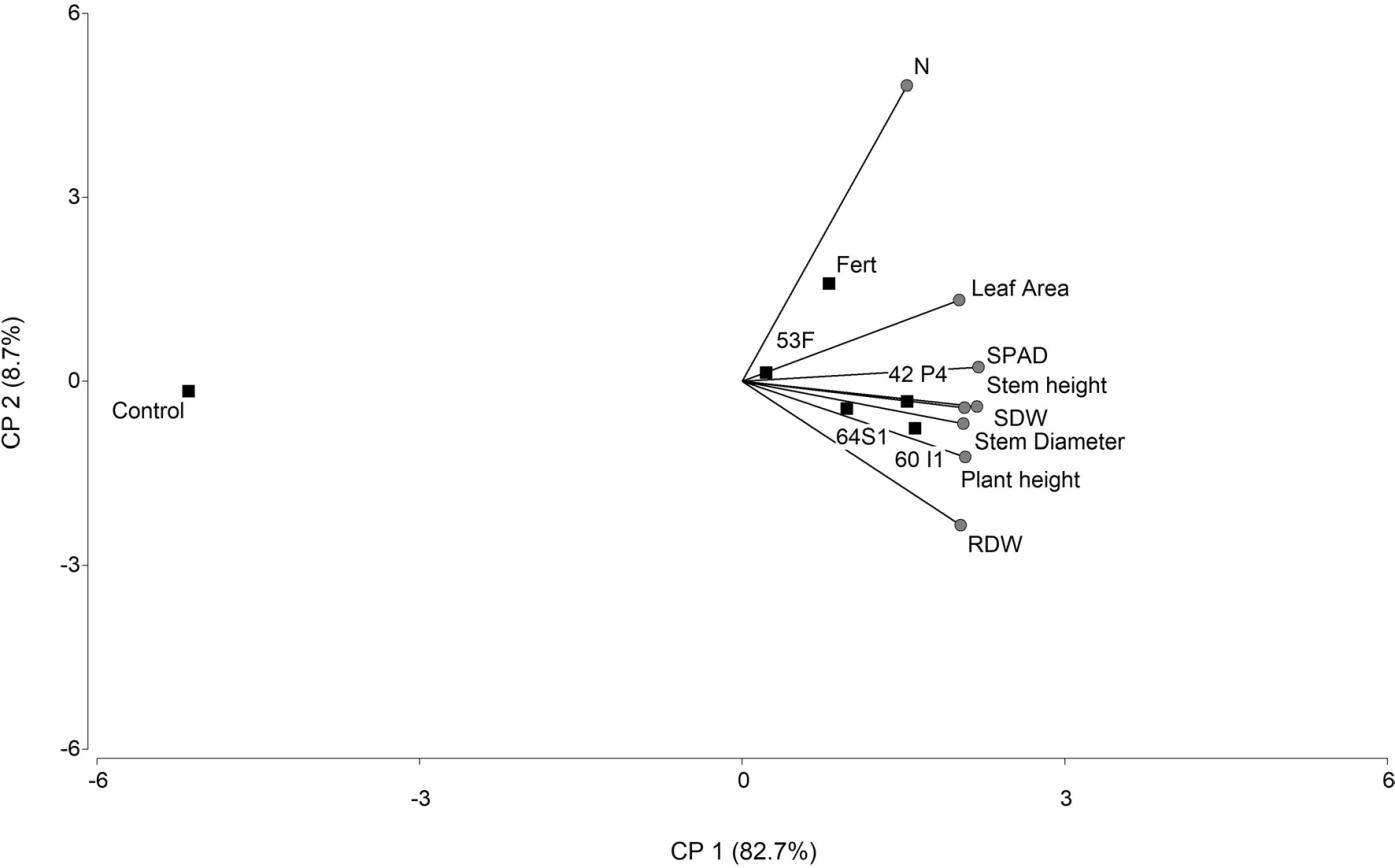
Biplot display of principal component analysis (PCA) of the parameters analyzed in tomato plantlets. Treatments: non-inoculated (Control), fertilized (Fert) and inoculated with: 64S1: *Enterobacter* 64S1, 42P4: *Pseudomonas* 42P4, 60I1: *Cellulosimicrobium* 60I1, 53F: *Ochrobactrum* 53F. Factors: Root dry weight (RDW), shoot dry weight (SDW), plant height, stem height, stem diameter, leaf area, nitrogen content (N) and SPAD index.

The growth parameters of UCO 14 seedlings treated with all of the bacterial suspensions were improved compared to the control; furthermore, growth parameters were equalled or improved those chemically fertilized (Fig. 3). The increase in root dry weigth (RDW) was 88, 74, 86 and 49% higher with *Enterobacter* 64S1, *Pseudomonas* 42P4, *Cellulosimicrobium* 60I1, *Ochrobactrum* 53F, respectively with respect to non-inoculated plants. The inoculation with *Ochrobactrum* 53F equaled this parameter value with those plants chemically fertilized (Fig. 3a). Moreover, 64S1, 60I1 and 42P4 strains increased RDW more than fertilization treatment (27, 25 and 17% respectively). The shoot dry weigth (SDW) was increased by treatments with all of the strains (39–55%) compared to control and equaled the fertilization treatment. The major increase was registered with 60I1 strain, 55% over control treatment (Fig. 3b). Stem height, plant height, and leaf area (LA) increased after treatment with all strains by 8–13%, 8–13% and 22–31% respectively (Fig. 3c–e respectively). Similar results occur for stem diameter; with the exception of 53F treatment that not differ from control (Fig. 3f). The SPAD index and N content showed the same tendency that the parameters evaluated previously (with significant difference respect the control), and the chlorophylls and carotenoids content were also increased with the strains selected (Table 5). The principal component analysis (PCA) showed that inoculation and fertilization treatments were different from the control with 82.7% of variability explained by stem diameter, RDW, SDW, LA, stem and plant height, N content and SPAD index (Fig. 4).

**Table 5:** SPAD index, nitrogen content, chlorophyll a (Chl a), chlorophyll b (Chl b), total chlorophyll and carotenoids of tomato plants of the industrial variety UCO 14.

Treatments	SPAD index^†^	Nitrogen (mg plant^−1^)^†^	Chl a (μg cm^−2^ leaf)^†^	Chl b (μg cm^−2^ leaf)^†^	Total Chl (μg cm^−2^ leaf)^†^	Carotenoids (μg cm^−2^ leaf)^†^
Control	36.79 ± 0.53^b^	1.42 ± 0.02^c^	3.76 ± 0.27^b^	1.24 ± 0.09^b^	4.99 ± 0.36^b^	0.73 ± 0.04 ^b^
Fertilized	41.64 ± 0.30^a^	3.36 ± 0.24^a^	5.02 ± 0.41^a^	1.62 ± 0.11^a^	6.64 ± 0.52^a^	0.95 ± 0.06 ^a^
64S1	42.02 ± 0.48^a^	2.32 ± 0.06^b^	5.05 ± 0.20^a^	1.58 ± 0.06^a^	6.62 ± 0.26^a^	0.93 ± 0.03 ^a^
42P4	41.65 ± 0.28^a^	2.57 ± 0.11^b^	4.78 ± 0.02^a^	1.52 ± 0.03^a^	6.32 ± 0.16^a^	0.90 ± 0.02 ^a^
60I1	42.06 ± 0.36^a^	2.15 ± 0.20^b^	4.80 ± 0.29^a^	1.51 ± 0.07^a^	6.31 ± 0.36^a^	0.90 ± 0.03^a^
53F	41.61 ± 0.42^a^	2.25 ± 0.03^b^	4.86 ± 0.31^a^	1.54 ± 0.10^a^	6.41 ± 0.41^a^	0.91 ± 0.04^a^

^†^Values are means ± SE (n = 6). Statistical comparisons are among treatments within a single column. The different letters indicate significant differences according to LSD Fisher test (*P* < 0.05).

## Discussion

This study had the purpose to isolate and characterize native beneficial bacteria strains from Mendoza with the potential to enhance tomato plant performance. The isolation, identification and characterization of native soil bacteria are fundamental to select potential PGPR adapted to soil and edaphic conditions from Cuyo Region, Argentina. For that, 90 bacteria from roots and rhizosphere of industrial tomato cultivars were isolated and analyzed in their PGP capacities. Among them, 36 strains showed N_2_ fixation faculty (40% of the isolates). The majority of them showed phosphate solubilization ability and capacity to produce siderophores. These are important PGPR attributes. The most efficient strains were: 64S1, 42P4, 60I1 and 53F. Also, other in vitro PGP activities were tested and the advantage of tomato seedling inoculation with them individually was evaluated. The strains were identified as *Enterobacter* 64S1, *Pseudomonas* 42P4; *Cellulosimicrobium* 60I1 and *Ochrobactrum* 53F.

These 4 strains have potential as plant growth promoting bacteria either directly or indirectly. All strains produce ammonia, siderophores and the strains *Pseudomonas* 42P4, *Ochrobactrum* 53F and *Enterobacter* 64S1 show also protease activity. Siderophores deprive pathogens microorganism of iron^27^ and are efficient carriers of iron to plants^28^. The siderophores and proteases contributed to pathogen growth inhibition. Proteases enzymes catalyse the degradation of organic matter and thus are important for soil fertilization and degradation of fungal cell wall^29^. The cell growths vary among the strains, where the fastest growth was observed in *Enterobacter* 64S1 culture and the slowest in *Cellulosimicrobium* 60I1. Then, bacterial growth was tested at different temperatures in order to evaluate the bacteria capacity to sort events of temperature changes at field levels. In the Cuyo Region, the seedlings transplanted can be damaged by late-frost or by Zonda-wind (warm strong and very dry wind similar to Föhn wind). Zonda wind can raise temperatures as much as 14° C in a matter of hours and the wind event is often followed by a freezing cold front. The results showed that the isolates could tolerate events with temperatures between 4 °C–40 °C. All strains produce IAA, however, the highest concentration of IAA was found in *Enterobacter* 64S1 and the lowest in *Stenotrophomonas* 25X1culture. Production of the phytohormone IAA is widespread among bacteria inhabiting plants rhizosphere, and is produced via several different biosynthesis pathways, with a single bacterial strain sometimes containing more than one pathway^30^. IAA, which is a growth promoting phytohormone, has many functions such as affecting root development and lateral and adventitious root formation^31^.

In Mendoza the majority of the soils are saturated with calcium, in this alkaline calcareous soils, the ions of Ca reacts with phosphate ions and decreases P availability by forming insoluble phosphate compound such as tricalcium phosphate^32, 33^. The mono valent calcium phosphate is a soluble form readily available for plant growth. However, the simple compounds of calcium as mono and divalent phosphates are present in extremely small quantities because they are easily revert to the more insoluble forms^34^. In this contex, we analyzed the phosphate solubilization capacity of selected bacteria in liquid NBRIP-BPB medium containing Ca_3_(PO_4_)_2_ as insoluble phosphate source. *Enterobacter* 64S1 and *Pseudomonas* 42P4 strains produced a higher level of soluble phosphorus respect to *Ochrobactrum* 53F or *Cellulosimicrobium* 60I1. The two first strains showed the maximum levels of soluble Pr at 24 h and it was maintained until 7 days, whereas in *Ochrobactrum* 53F and *Cellulosimicrobium* 60I1 reached maximum value at 7 days. Considering these results and the reported by Collavino et al.^35^, 64S1 and 42P4 are considered “early solubilizers” and *Ochrobactrum* 53F and *Cellulosimicrobium* 60I1, “late solubilizers”. Thus, the bacterial kinetics and the ability to solubilize Ca_3_(PO4)_2_ were different for each strain, in agreement with Anzuay et al.^36^. The four strains presented high viability at 7 days and decreased culture medium pH, indicating that acidification of the medium could be related to P solubilization. The amount of soluble phosphorus produced by *Enterobacter* 64S1 was minor to those reported for *P. fluorescens* PM T1 strain^13^ but presented intermediate values with respect to others PGPR strains. These results suggest that *Enterobacter* 64S1 and *Pseudomonas* 42P4 are promising phosphate solubilizing bacteria for Mendoza region considering the alkaline calcareous nature of soils. Furthermore, it was of interest to study the ability of these strains to solubilize more insoluble inorganic P sources, considering Bashan et al.^37^ that reported that the study of only one metal-P source is not suitable as an universal selection factor for phosphate solubilizing bacteria. In this sense, the ability of *Enterobacter* 64S1 and *Pseudomonas* 42P4 strains to solubilize other insoluble inorganic P sources (FePO_4_ and AlPO_4_) were also evaluated and our data showed that both strains have this ability. The Pr in NBRIP-BPB medium containing AlPO_4_ was higher than those observed with FePO_4_. Similar results were found by several authors when analyzing phosphate solubilizing ability^38^. The level of soluble Pr in NBRIP-BPB medium with AlPO_4_ was threefold more with *Enterobacter* 64S1 whereas in NBRIP-BPB medium with FePO4 was similar in both strains, demonstrating the efficient capacity of phosphate solubilization. The pH in the medium containing either Al or Fe for both strains decreased to 3.5–3.6, and the CFU value reached 10^5–6^ CFU mL^−1^. In this study, we found that amounts of soluble Pr by each strain in liquid medium showed correlation with the diameter of halo of clearance produced in solid NBRIP medium. Nevertheless, others studies considered that halo formation is not a suitable criterion for selection of efficient phosphate solubilizers^35^. The higher level of soluble Pr was found in Gram-negative bacteria (75%) consistent with other authors^13, 35, 36^. The mineral phosphate solubilization is accompanied by a decrease in pH and one of the most accepted mechanisms is the production of different organic acids, mainly gluconate. The production of this acid which is responsible of mineral phosphate solubilizing phenotype^39^ has been reported in *Pseudomonas, Enterobacter, Pantoea, Serratia* and others PGPR^36^.

The *pqq* gene encoding PQQ is involved in phosphorus solubilization as a co-factor in extracellular oxidation of glucose to gluconic acid by a glucose dehydrogenase^14^. The detection of *pqq*E gene in *Enterobacter* 64S1 and *Pseudomonas* 42P4 suggests that these bacteria produce PQQ and thus that gluconic acid is produced by them. The fact that *pqq*E gene was not amplified from the DNA of some isolates analyzed, may be due to a failure in the designed primers, in the step of primers annealing, or that a different gluconic acid pathway could be operating in these bacteria.

Plants assays indicated that PGPR treatment increased all of the morphological parameters evaluated, confirming the ability of the strains to promote tomato growth. The parameters increased by inoculation were: RDW, SDW, stem height, plant height, LA and stem diameter of tomato plantlets. These results were similar to chemical fertilization with exception of RDW in which the inoculation with *Enterobacter* 64S1, *Pseudomonas* 42P4 and *Cellulosimicrobium* 60I1 overcame the fertilization. In the principal component graph, the control treatment was separated from inoculation and fertilization treatment, and the treatments with 64S1, 42P4 and 60I1 were grouped.

The effects on roots can be explained through the production of IAA by these isolated. At relatively high concentrations, natural auxins stimulate shoot elongation and root induction while reducing root elongation^40^. Several works have reported that the synthesis of IAA is often associated with plant growth stimulation by microorganisms, including *Pseudomonas putida*^41, 42^. On tomato plants, it was found that the synthesis of IAA through tryptophan-dependent pathways by PGPR such as *P. putida* or *Trichoderma atroviride* affected the growth of the tomato seedlings^43^. A greater development of the radical system, due to exogenous sources of IAA, could cause changes in the morphology of the root system, which could influence the uptake of nutrients by the plant^44^. In tomato plant development, the critical period of nutrient absorption occurs during the first week of growth^22^. As these strains are able to fix N_2_, produce ammonium and solubilize insoluble phosphates, the plant can absorb these macronutrients and, as consequence, plant growth was increased. Inoculation with bacterial isolates increased plantlet N-aerial content compared to the control plants. Considering the results presented, the inoculation with the native selected bacterial increased the tomato plantlets quality, mainly by enhancing root development and increasing stem diameter, characters that are usually correlated with transplant vigor, a higher survival rate and growth after transplanting^45^. Furthermore, inoculation effects were equal or higher than the ones obtained by synthetic fertilization. In addition, the enhanced total chlorophyll content in leaves of inoculated plantlets may contribute to a better plant growth.

## Conclusion

In this study, the ability to promote plant growth of four strains selected from a natural environment and identified as *Pseudomonas* 42P4; *Ochrobactrum* 53F; *Cellulosimicrobium* 60I1; *Enterobacter* 64S1 on tomato plantlets was demonstrated. The strains had the ability to produce IAA, siderophores, nitrogen fixation and solubilization of different sources of P. Therefore, these traits positively influence overall plant growth and root development enhancing nutrient uptake. The strains more effective were *Pseudomonas* 42P4 and *Enterobacter* 64S1 both in vitro and in vivo assays*.* This study shows that the inoculation with the native selected bacterial increased the quality of tomato plantlets. However, future studies will be required to investigate these isolates under different field conditions and to assess their potential as bioinoculants in agriculture.

## Materials and methods

### Bacteria isolation

Bacteria associated with tomato crops were isolated from rhizospheric soil and roots of *Solanum lycopersicum* cv. UCO 14 plants growing in a field located in Maipú (latitude 32°56′41ʺ S, longitude 68°40′31ʺ W, altitude 677 m a.s.l.) Mendoza, Argentina. Roots and soil samples were collected at a root depth of 0–5 cm and 5–15 cm. Roots were submerged in 50 mL of sterile phosphate buffer (PBS^46^) and the supernatants were serially diluted from 10^–1^ to 10^–5^. These tubes were named as rhizosphere samples. The root´s surface was disinfected with 70% ethanol for 1 min and washed several times with sterile distilled water. Then, roots were cut into pieces and 1 g of each one was macerated in PBS. Serial dilutions (10 X) were made and aliquots of 0.1 mL from decimal dilution (10^–4^–10^–6^) were plated in Petri dishes with bacterial Luria Broth Base (Miller’s LB Broth Base Invitrogen, Buenos Aires, Argentina) medium^16^. After being incubated for 72 h at 28 °C, colonies were sub-cultured in LB medium and it grouped according to phenotypic characteristics and Gram stain reaction (Gram Britania, Buenos Aires, Argentina) and subjected to further characterization. The isolates were maintained in LB medium with 20% glycerol on micro-tubes at −20 °C.

### Biological nitrogen fixation ability

Bacterial ability to fix N_2_ was determined in vials with N free semisolid medium (NFb^47^). Bacteria grew in liquid LB medium during 24 h at 28 °C and 120 rpm (Shaker Pro, Viking, BIO-CONTROL, Buenos Aires, Argentina). Then, 1 mL of bacterial culture was centrifuged 2 min at 3,000 rpm, supernatant was removed and the pellet resuspended in physiological solution (0.85% NaCl). The last procedure was repeated twice. Aliquots (50 μL) of the bacterial suspension were inoculated into vials containing 5 mL of NFb medium. They were incubated for 7 days at 28 °C. The formation of a pellicle was considered as positive, indicating the bacteria’s ability to fix N_2_.

### Phosphate solubilization ability

In vitro inorganic phosphate-solubilizing ability was determined in NBRIP-BPB solid medium (National Botanical Research Institute’s phosphate grown medium^48^) that contains Ca_3_(PO4)_2_ (5 g L^−1^) as the only P source. The ability to solubilize other inorganic insoluble phosphate sources as FePO_4_ (1 g L^−1^) and AlPO_4_ (2 g L^−1^) was determined by replacing tricalcium phosphate for these P sources. Fresh bacterial cultures (10 μL, 10^8^ CFU mL^−1^) were spotted on plates containing NBRIP-BPB medium and incubated at 28 °C for 7 days. The halo of clearance around the bacterial colony indicated phosphate solubilization ability. The solubilization zone and colony diameter were measured and solubilization efficiency was evaluated according to Dawwam et al.^49^ with the following equation:

1 $${\text{SE}}\, = \,\left( {{\text{solubilization diameter}}/{\text{growth diameter}}} \right)\, \times \,{1}00$$

### Siderophores production

Siderophores production was screened using the Chrome Azurol S-agar (CAS-agar) protocol according to Milagres et al.^50^ modified by Pinter et al.^51^. Plates of 5 cm in diameter with a basal layer of blue CAS-agar (3.5 mL), and a top layer of LB-agar (4 mL) were prepared. Aliquots (10 μL) of the bacterial suspension (obtained as described above) were seeded on the plates and incubated for 7 days at 28 °C. The appearance of an orange halo in the CAS-agar was evaluated. The percentage of halo was determined according to Pinter et al.^51^ by the equation:

2 $${\text{Percentage of halo}}\, = \,\left( {{\text{halo diameter}}-{\text{colony diameter}}} \right) \, /{\text{ colony diameter}}$$

### Characterization of selected bacterial isolates

#### Biochemical characterization

According to the ability to fix N_2_, solubilize phosphates and production of siderophores were selected the 5 most promising bacteria (64S1, 53F, 25X1, 42P4 and 60I1). The catalase activity was determined transferring 400 μL of bacterial suspension to the surface of a clean dry glass slide. Then, a drop of 1.5% H_2_O_2_ was placed on the slide and mixed. A positive result was the production of O_2_ evidenced by bubbling^16^.

The presence of cytochrome oxidase enzyme was measured with paper discs impregnated with N,N-dimethyl-p-phenylenediamine and α-naphthol reaction to indophenol blue (Sigma-Aldrich, Argentina). The enzyme activity was evidence by the indophenol blue product^16^.

Protease activity was determined on 3% (w v^−1^) powdered milk-agar plates according to Walsh et al.^52^.

Bacterial ability to produce ammonia was checked according to Cappuccino et al.^53^. Isolate was inoculated in 10 mL peptone broth and incubated at 28 °C for 48 h at 120 rpm. After incubation 0.5 mL of Nessler’s reagent was added to the tube. The development of faint yellow to dark brown color indicates the ammonia production.

The growth rate was monitored by spectrophotometric absorbance at 530 nm (OD_530_, biomass production) in UV–Vis spectrophotometer Cary 50 (Varian Inc., Mulgrave, Palo Alto, CA, USA). For that one colony of the strains were cultured on LB medium in an orbital shaker (Shaker Pro, Viking, BIO-CONTROL, Buenos Aires, Argentina) at 28 °C and 120 rpm for over 12 h. Then, 500 µL of each bacterial culture was transferred to different erlenmeyer with 50 mL LB medium and the growth was evaluated by OD_530_ every 2 h until the stationary phase.

The influence of temperature on the strains selected (64S1, 53F, 25X1, 42P4 and 60I1) was evaluated. Each culture started from a pre-cultured as mentioned previously. Then, 10 µL of each bacterial culture was transferred to different erlenmeyer with 10 mL LB medium for 24 h at 4 °C, 28 °C or 40 °C. Then, the colony forming units (CFU) mL^−1^ for each treatment were determined.

Indole-3-acetic acid (IAA) production was determined by gas chromatography- mass spectrometry (GC–MS). Each strain was cultivated in 250 mL Erlenmeyer flasks containing 50 mL of NFb medium plus NH_4_Cl (1.25 g L^−1^) as N source. The flasks were incubated in an orbital shaking (Shaker Pro, Viking, BIO-CONTROL, Buenos Aires, Argentina) in darkness at 120 rpm and 28 °C, until stationary phase as determined by OD_530_ (biomass production), and further processed as previously described in Cohen et al.^11^ with modifications. The bacterial cultures were sonicated twice for 5 min and centrifuged 10 min at 8,000 rpm and 4 °C. The cells were discarded, the supernatant was adjusted to pH 3.0 with acetic acid and partitioned 3 times with an equal volume of ethyl acetate (saturated with 1% acetic acid), pH 3.0. The ethyl acetate fraction was evaporated in a rotary evaporator under vacuum at 35 °C. Then, the content of the free form of IAA was analyzed according to Podda et al.^54^. Briefly, the dried sample was resuspended with 1 mL isopropanol:acetic acid (95:5, v/v), to which 500 ng of ^13^C_6_-IAA (OlChemIm, Olomouc, Czech Republic) were added as internal standard for quantitative mass-spectral analysis. After overnight isotope equilibration at 4 °C, the solution was centrifuged and evaporated to dryness, and the residues were taken up with 300 μL methanol and methylated using diazomethane, then dried under a gentle N_2_ gas stream^55^. The samples were finally resuspended in 30 μL ethyl acetate and 2 μL were injected into a GC–MS system (7890A-5975C, Agilent Technologies, Santa Clara, CA, USA). Ions monitored were: m/z 130, 136 for the base peak (quinolinium ion) and m/z 189, 195 for the molecular ion of the methyl-IAA and the methyl-13C_6_-IAA, respectively. For absolute quantification, the endogenous hormone levels were estimated from the corresponding peak area by calculating the ratios between m/z 130/136 and m/z 189/195 according to the principles of isotope dilution^56^. The amounts of free IAA were calculated from three replicated measurements.

#### Molecular identification

The selected strains: 64S1, 53F, 25X1, 42P4 and 60I1 were identified by 16S rRNA partial gene sequencing, phylogenetic analysis and has been deposited in GenBank data bank (NCBI) under accession numbers MT047267, MT047264, MT044591, MT045593 and MT047266 respectively. Genomic DNA was extracted from 1 mL of each one bacterial culture in early stationary phase. The extraction was performed with the commercial kit QIAamp, DNA mini kit (Qiagen, Hilden, Germany) following the manufacturer’s instructions. Each bacterial strain was amplified using 27F forward primer (5′-AGAGTTTGATC(AC)TGGCTCAG-3′) and 1492R reverse primer (5′- CGG(CT)TACCTTGTTACGACTT-3′). PCR reaction was carried out in 20 µL reaction volumes containing 50 ng of template DNA, 20 pmol of each primer, 0.2 mM dNTPs and 1 U Taq polymerase in PCR buffer. Reactions were cycled 35 times in a thermocycler (T-Professional Basic Biometra, Germany); the steps were: 94 °C for 30 s, 58 °C for 30 s, 72 °C for 90 s followed by a final extension at 72 °C for 10 min. Amplified PCR products were checked and separated by gel electrophoresis on 1% (w/v) agarose gel. Bands were cleaned with PCR Purification Kit (PureLink, Invitrogen, Germany) to follow sequencing. The PCR reactions were sequenced by Macrogen (Korea). The 16 s rDNA sequences were analyzed with Bioedit Sequence Alignment editor 5.0.3, checked manually and corrected if necessary. BLAST analysis was performed to compare the sequences obtained with the data available in NCBI. Maximum likelihood analyses were performed with Mega-X under the General Time Reversible model with parameters for invariant sites and gamma-distributed rate heterogeneity (4 categories). One hundred bootstrap replicates were performed.

#### Quantification of soluble P released from Ca_3_(PO_4_)_2_, FePO_4_ and AlPO_4_ sources

The soluble P released (Pr) into the NBRIP-BPB liquid culture medium containing Ca_3_(PO_4_)_2,_ FePO_4_ and AlPO_4_) was determined following Fiske and Subbarow^57^ method with modifications. For it, 100 µL of an overnight inoculum (approximately 10^8^ CFU mL^−1^) in LB medium of each bacterium was transferred to 15 mL of NBRIP-BPB medium containing Ca_3_(PO4)_2_ (5 g L^−1^), FePO_4_ (1 g L^−1^) or AlPO_4_ (2 g L^−1^). After 24, 48, 72 and 168 h of growth, 1.5 mL of bacterial culture were sampled and centrifuged for 12 min at 10.000 rpm. The amount of soluble Pr to the medium was quantified spectrophotometrically by measuring absorbance at 660 nm (OD_660_)^36^. Also, the CFU mL^−1^ and supernatant pH of each sample were measured.

#### Amplification of pqqE gene

Total bacterial DNA was obtained by using the procedure described by Walsh et al.^58^. The amplification of a 700 bp *pqq*E gene fragment was assayed using the primers F317 and R1019^36^. Moreover, the amplification of a ~ 350 bp *pqq*E gene fragment was analyzed using the primers *pqq*EENT1-(5ʹCCGAACAGTGGATTGAGGTT3ʹ) and *pqq*EENT2-(5ʹAATTCAGCACCATCGGGTAG3ʹ) designed for this study by multiple alignment of gene *pqq*E sequences obtained from NCBI gene data bank of bacteria belonging to the genera: *Pantoea, Enterobacter, Klebsiella, Serratia and Erwinia.* Each PCR reaction (20 μL) contained 2 μL (10 μM) of each primer, 2 μL (10X) of buffer, 2 μL (2 mM) of dNTPs, 7.4 μL of sterile bidistilled water, 2 μL of MgCl_2_ (50 mM), 0.2 μL of Taq DNA polymerase (5 U μL ^− 1^) and 2.4 μL of template DNA. Amplifications were performed in a DNA thermal cycler (Mastercycler Eppendorf). The temperature profile for PCR-*pqq*E was: An initial cycle at 95 °C for 1 min, followed by 35 cycles at 94 °C for 1 min, at 55 °C for 1 min and at 72 °C for 2 min, and finally 72 °C for 10 min. Then, 10 μL PCR products were separated by horizontal electrophoresis on 1.2% (w v^−1^) agarose gels stained with SYBR Green II (Molecular Probes).

### Plant materials, growth conditions and seedling inoculation

#### Bacterial cultures

One colony of each strain selected was pre-cultured in LB medium as mentioned previously until reaching a concentration of 10^8^ CFU mL^−1^. Bacteria cultures were centrifuged at 7,300 rpm for 10 min at 4 °C. The supernatants were discarded and the pellets were washed with sterile PBS, centrifuged again, and diluted to 10^7^ CFU mL^−1^ of PBS buffer for further inoculation.

A growth chamber experiment was conducted to test the effect of selected PGPR on tomato plantlets. A tomato (*Solanum lycopersicum* L) industrial variety UCO 14 (INTA, Mendoza, Argentina) was used in this study. Tomato seeds were surface sterilized with 70% ethanol for 1 min, followed by rinsing with sterile distilled water. Then, they were sown in 60 mL alveolar boxes on sterilized soil. The growth medium Kekkilä DSM 1 W (Kekkilä professional, Vantaa, Finland) contained 70% brown and 30% dark *Sphagnum fuscum* dominant peat, N–P_2_O_5_–K_2_O 15–12–29 and microelements 0.6 kg m^−3^, pH 5.9, electrical conductivity 0.2 dS m^−1^. A minimal fertilization treatment with 1 mL solution of 10.5 g L^−1^with Hakaphos Base 18–18–18 (COMPO, Spain) was applied to each alveolar box. After seeding, water was applied daily to keep the soil water status close to field capacity. The plants were cultured with a photoperiod of 16 h of cool white light (100 μmol m^−2^ s^−1^) at a temperature of 22 ± 2 °C. Fifteen days after sown, the following treatments were applied: (1) Control (C): 1 mL PBS; (2) *Cellulosimicrobium* 60I1: 1 mL PBS with 10^7^ CFU mL^−1^; (3) *Ochrobactrum* 53F: 1 mL PBS containing 10^7^ CFU mL^−1^; (4) *Enterobacter* 64S1 : 1 mL PBS with 10^7^ CFU mL^−1^; (5) *Pseudomonas* 42P4: 1 mL PBS with 10^7^ CFU mL^−1^; (6) Fertilizer: 1 mL solution of 10.5 g L^−1^ Hakaphos Base 18:18:18 (COMPO, Spain). All treatments were applied on the soil surface. The experimental design consisted of 6 treatments with 18 replicates (n = 18). Six seedlings were used to determine photosynthetic pigments, and 11 were randomly chosen to measure the other parameters. Plants in alveolar boxes were placed in trays in a completely randomized design and they were rotated weekly. This experiment was repeated twice and the magnitudes of the responses were similar each time.

#### Growth parameters

After 25 days of plant inoculation, plants were carefully removed from the soil medium. Roots were washed in slow running water to remove adhering soil and dried with a paper towel to remove water excess. Plant height, stem height (from the base to the last branch) and basal diameter were determined. Leaf area (LA), was determined using the software Image J. Root and shoot dry weights (RDW and SDW respectively) were determined after drying the samples for 7 days in the hot oven at 60 °C.

#### Photosynthetic pigments and nitrogen content

The relative chlorophyll content of young leaves was measured using a portable chlorophyll meter (SPAD-502, Konica Minolta Sensing, Osaka, Japan). Pigment determinations were done spectrophotometrically according to Cohen et al.^17^. Total chlorophyll (Chl; Chl a + Chl b) and carotenoid levels were measured from 1 cm^2^ leaf. Aerial N content was determined by Kjeldahl method according to Nelson and Sommers^59^.

### Statistical analysis

Statistical analysis was performed with the one-way ANOVA and Fisher’s multiple tests to discriminate between the averages by the minimum difference with a significance level of *P* ≤ 0.05 (Software InfoStat version 2017; Grupo InfoStat, FCA, Universidad Nacional de Córdoba, Argentina). Principal component analyses (PCA) are presented as Biplot graphs.

## Supplementary information

Supplementary file1

## Data Availability

The sequencing data generated and analyzed during the current study are available in the National Center for Biotechnology Information (NCBI), U.S. repository under the accession numbers MT047267, MT047264, MT044591, MT045593 and MT047266.
