# Supplementary material for: Native bacteria isolated from roots and rhizosphere of Solanum lycopersicum L. increase tomato seedling growth under a reduced fertilization regime
Source: Sci Rep. 2020 Sep 24;10:15642. doi: 10.1038/s41598-020-72507-4 (PMC7515909; doi:10.1038/s41598-020-72507-4)
Supplement: Supplementary file 1 — Supplementary file1 [file 41598_2020_72507_MOESM1_ESM.docx]

**Native bacteria isolated from roots and rhizosphere of *Solanum lycopersicum L*. increase tomato seedling growth under a reduced fertilization regime**

María Micaela Pérez-Rodriguez^a^, Patricia Piccoli^a^, María Soledad Anzuay^b^, Rita Baraldi^c^, Luisa Neri^c^, Tania Taurian^b^, Miguel Andrés Lobato Ureche^a^, Diana María Segura^a^, Ana Carmen Cohen^a*^.


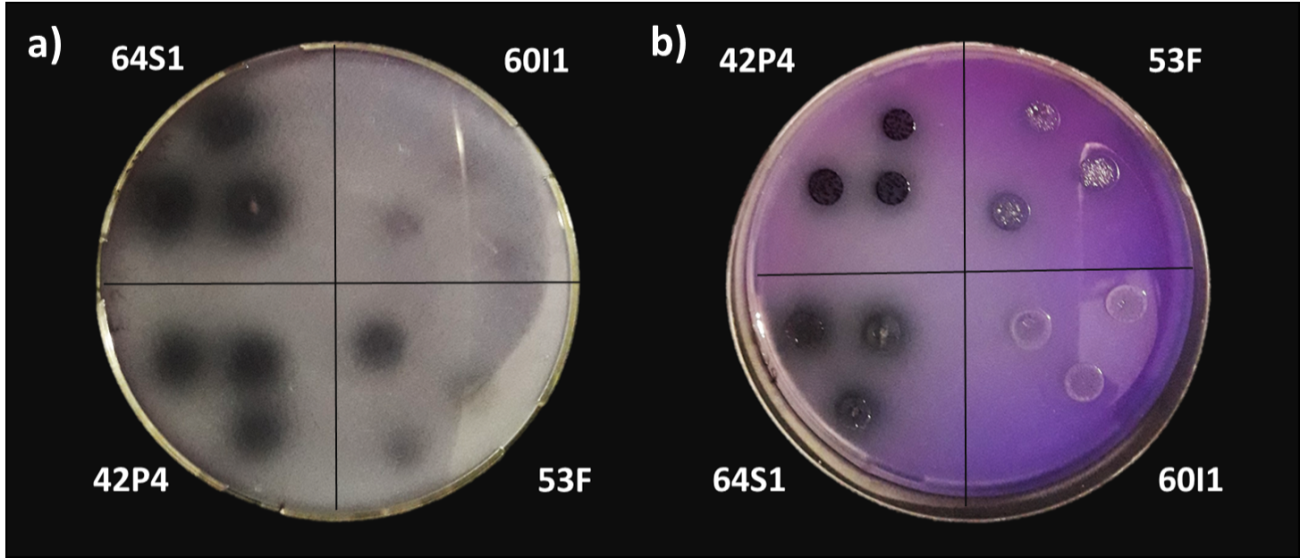


**Supplementary Figure S1.** a) bottom and b) top view of phosphate solubilization halo produced by 64S1, 60I1, 42P4 and 53F strains isolated from rhizosphere and roots of tomato crop.


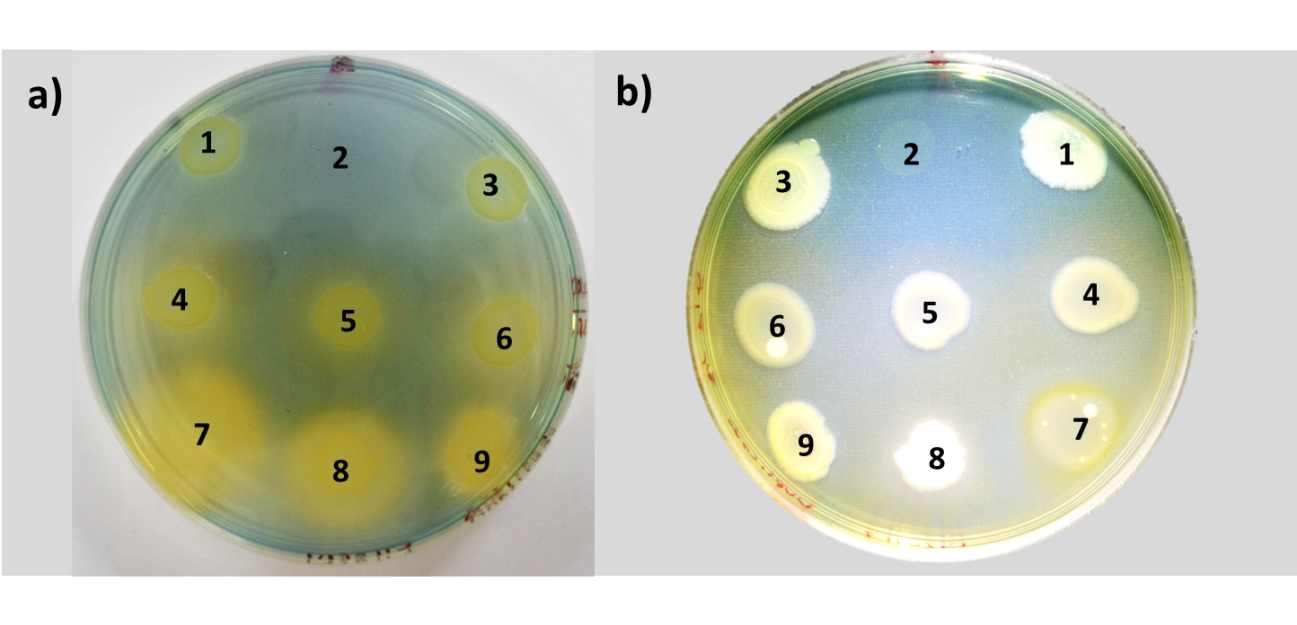


**Supplementary Figure S2.** a) bottom and b) top view of siderophore production by different strains isolated from rhizosphere and roots of tomato crop. **1)** 65I4, **2)** 6L, **3)** 59U5, **4)** 60I1, **5)** 53F, **6)** 27T4, **7)** 42P4, **8)** 25X1, **9)** 64S1.





**Supplementary Figure S3.** Cell growth (DO_530_) of each strain cultivated for 30 h at 28 °C on LB medium as described in Materials and Methods. Each point represents an average of three independent experiments with standard error.


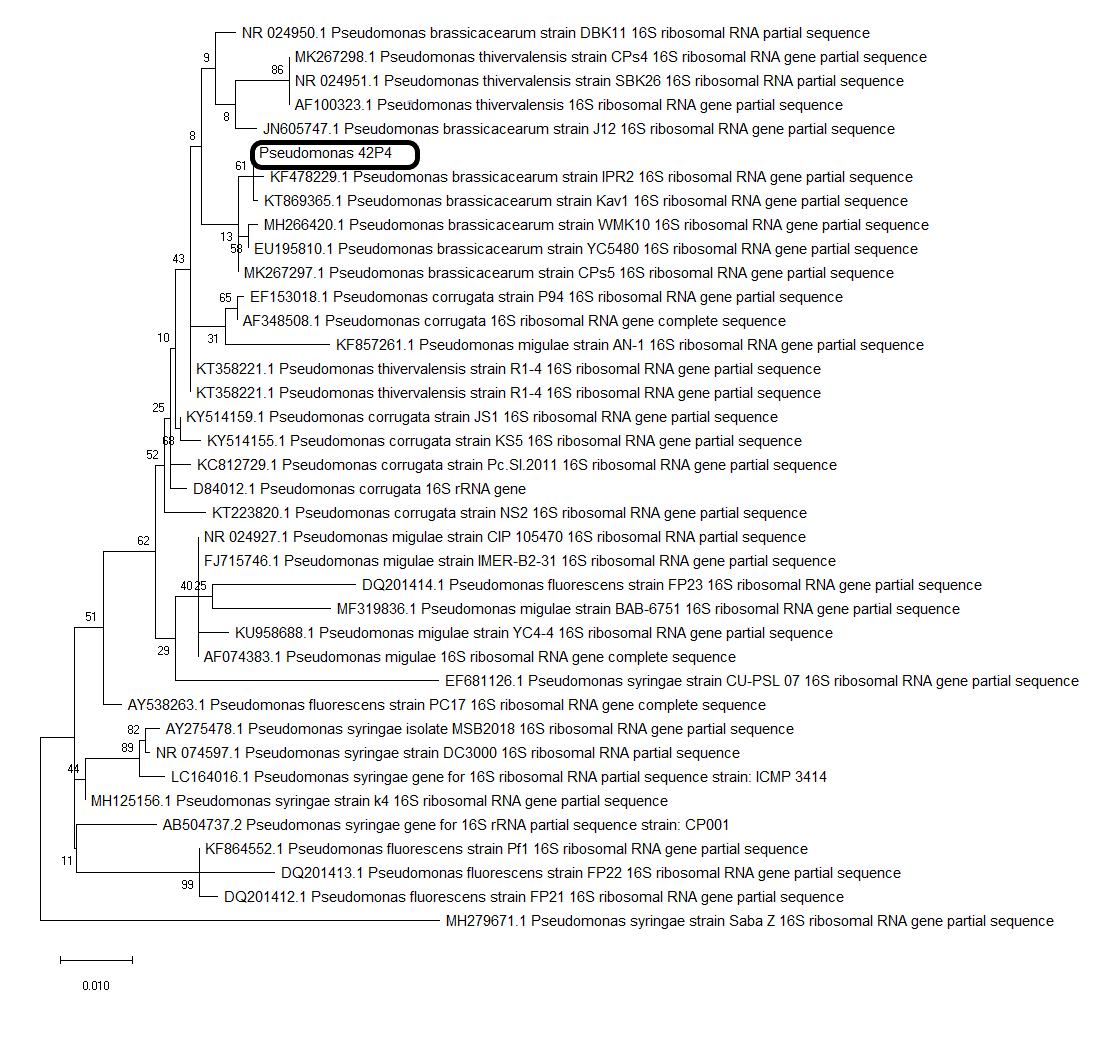


**Supplementary Figure S4.** Phylogenetic tree between *Pseudomonas* 42P4 and its phylogenetically closest microorganisms based on the 16S rDNA using the maximum likelihood method, MEGA-X.
